# A Mobile Phone App to Improve the Mental Health of Taxi Drivers: Single-Arm Feasibility Trial

**DOI:** 10.2196/13133

**Published:** 2020-01-15

**Authors:** Sandra Davidson, Susan Fletcher, Greg Wadley, Nicola Reavley, Jane Gunn, Darryl Wade

**Affiliations:** 1 Department of General Practice The University of Melbourne Carlton Australia; 2 School of Computing and Information Systems The University of Melbourne Parkville Australia; 3 Melbourne School of Population and Global Health The University of Melbourne Carlton Australia; 4 Centre for Posttraumatic Mental Health Phoenix Australia Carlton Australia; 5 Department of Psychiatry University of Melbourne Parkville Australia

**Keywords:** mental health, eHealth, taxi drivers, immigrant, help-seeking behavior, self-help

## Abstract

**Background:**

Psychological distress among taxi drivers is 5 times higher than that in the general population, and more than half of all drivers have experienced 3 or more potentially traumatic events in their lifetime. Nevertheless, help-seeking for mental health problems in this male-dominated, predominately immigrant workforce is low. Mobile technologies have the potential to increase mental health awareness, teach self-help skills, and encourage help-seeking in this hard-to-reach population.

**Objective:**

This study aimed to assess the feasibility, acceptability, and potential efficacy of *Driving to Health*, a mobile phone–friendly mental health website app designed for people working as taxi drivers.

**Methods:**

Drivers (n=46) were recruited from the Melbourne Airport Taxi Holding Yard to participate in a single-arm trial. Self-reported, paper-based assessments were completed at baseline and at 1 month. Feasibility was measured by completion rates, representativeness of study participants, and levels of use. Acceptability was assessed by measuring users’ perception of the quality of the app and anticipated levels of future use. The efficacy of *Driving to Health* to increase awareness, self-help behaviors, and intentions to seek help was assessed using the user version of the Mobile App Rating Scale (uMARS) and the General Help-Seeking Questionnaire (GHSQ). Psychological symptoms were measured using the short form of the Depression, Anxiety, and Stress Scale (DASS-21). Data were analyzed using complete case analysis.

**Results:**

In total, 42 participants comprising drivers from 10 different countries of origin, and 14 different languages, completed pre- and poststudy measures (42/46, 91% completion rate). Just under half (45%) of all users used the app more than once with an average visit of 4 min 8 seconds. Responding to the uMARS, 62% (26/42) of the participants said that they would recommend the app to many people. Nearly all (40/42, 95%) participants said that *Driving to Health* increased awareness of their own mental health; 86% (36/42) said that it increased their mental health knowledge; and 76% (32/42) said that it increased their self-help behaviors. Increases in help-seeking intentions on the GHSQ were not significant, and increases on all 3 scales of DASS-21 were not reliable or meaningful.

**Conclusions:**

This study suggests that *Driving to Health* is an acceptable and feasible electronic health intervention for a hard-to-reach population. Our findings also suggest that *Driving to Health* results in increases in mental health awareness, behaviors, and willingness to seek help.

## Introduction

### Background

The need for mental health support in high-risk industries, such as the military and emergency services, is well known. However, another large occupational group that has received relatively little attention is that of taxi drivers. A recent Australian study found that 61% of urban taxi drivers reported high or very high levels of psychological distress compared with just 12% of the general population [1]. The origin of such distress among taxi drivers is multifaceted and includes poor working conditions characterized by long hours, night shift, low pay, lack of physical activity, high rates of verbal assault and physical violence, and increased competition from ride-sharing services [2-6]. A recent spate of suicides among taxi drivers working in New York dramatically illustrates the vulnerability to mental health issues among taxi drivers [7]. Despite their substantial mental health needs, relatively few taxi drivers access health services, especially mental health services [1,8].

Nearly all taxi drivers are male, and over 90% of drivers working in urban areas are immigrants [1,9-11]. Previous studies show that immigrants tend to use health services less often than the locally born population, a phenomenon attributed to lower mental health knowledge and awareness, higher rates of mental health stigma, and difficulties in navigating an unfamiliar health system among people from culturally and linguistically diverse communities [12-16]. Lack of time may be an additional barrier to help-seeking for men working as taxi drivers who typically work long hours [1].

These long hours are characterized by extensive periods of waiting between jobs, and drivers can find themselves idle for up to 8 hours of a 12-hour shift. They pass this time using their mobile phone to socialize with friends, search the internet, and play games [17]. Our research shows that 97% of drivers use a mobile phone during their working day [1], which suggests that mobile phones may provide an avenue to address some of the disparities experienced by drivers in accessing mental health care [18-21].

Evidence suggests that individuals from minority populations access web-based health information at a greater rate than majority populations [22] and that electronic health (eHealth) interventions delivered via mobile technologies such as mobile phone apps and mobile-enabled websites can support skills acquisition and symptom monitoring [23], promote health behavior-change [24], and increase intentions to seek help from professional services [25]. Importantly for taxi drivers, mobile phone-delivered eHealth care can be accessed at any time and often at a relatively low cost, which is important for drivers on a low income who work long hours, often during the night when traditional health services are unavailable. Accessing eHealth services also offers anonymity, which may appeal to drivers whose cultural background r personal beliefs include a high degree of mental health stigma [22,26].

Clearly, taxi drivers are a hard-to-reach population for whom targeted mental health support is urgently needed. The synchronicity between drivers’ ownership and use of mobile phones and the utility of mobile phones in delivering health services make them an especially promising mode of mental health intervention for this hard-to-reach population [27].

### The Driving to Health App

Based on our research into the mental health needs and technology use among people working as taxi drivers [1,17], we designed and developed *Driving to Health*. *Driving to Health* is a self-guided, smartphone-friendly website app that can be used on both iOS (Apple) and Android (Google) mobile devices. The app was designed to provide taxi drivers with psychoeducation, self-help strategies, symptom assessment, and links to health professionals. Consistent with previous work on designing culturally relevant health apps for minority populations, the design phase was based on an iterative action process which included taxi drivers and a team of mental health experts comprising psychologists, primary care physicians, posttraumatic stress specialists, and human-computer interaction researchers [28].

The website underwent 2 rounds of user experience testing with taxi drivers, resulting in the version tested in this study. Designed with a culturally and linguistically diverse user group in mind, text in the website is limited, and most of the information is delivered via video demonstrations and audio explanations. All text and audio are presented in English. Local taxi drivers were employed to be the actors in the videos, and a professional voice actor was employed to provide the narration. All written information in the website has a Flesch readability score between 60 and 70 and a reading grade level between 7 and 8 [29,30].

**Figure 1:**
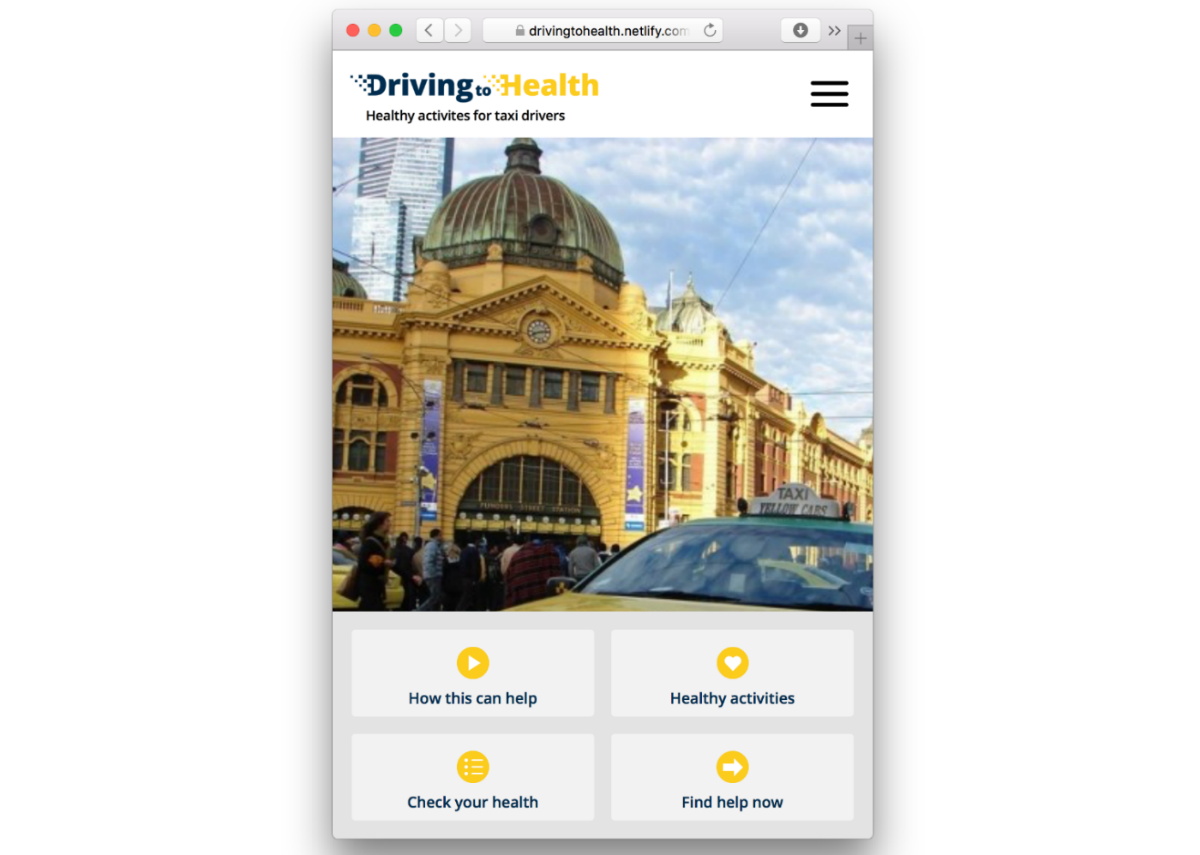
*Driving to Health* home screen.

**Figure 2:**
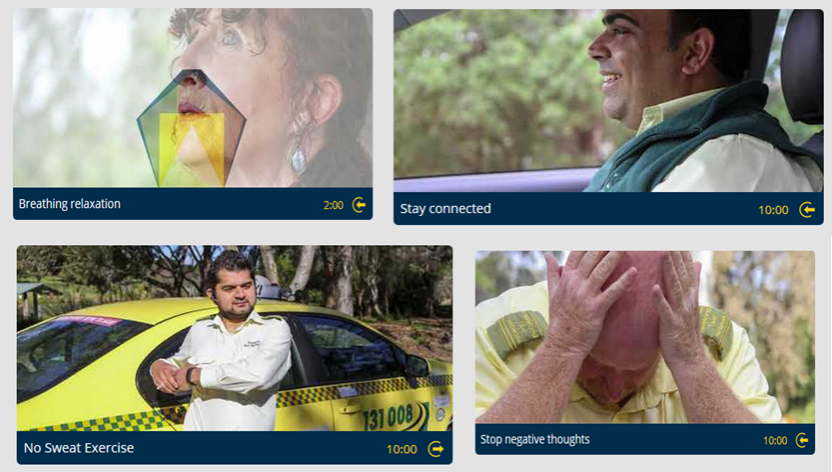
Examples of self-help activities in *Driving to Health*.

*Driving to Health* has 4 main components that can be accessed in any order from the home screen (Figure 1):

1. The How This Can Help component was designed to engage drivers by presenting images and words capturing the essence of what it is like to work as a taxi driver and to introduce users to the purpose of the tool.
2. Symptom awareness is facilitated by the Check Your Health component which includes the 10-item Kessler Psychological Distress scale (K10) [31] and psychoeducational information about stress, mental health disorders and treatment options. The K10 was selected as it provides a measure of general distress that is not tied to a particular disorder or classification system. At the same time, it is highly predictive of formal diagnosis and has excellent psychometric properties [31]. The K10 is widely used and validated in Australia and internationally in national health surveys [32]and is the most frequently used outcome measure in Australian primary health care [33], providing the potential for Driving to Health scores in the future to be used by health care providers to inform treatment and referral decisions. When users complete the K10, they are provided with feedback based on their score. For example, users who score in the high or very high range on the K10 are presented with feedback that they may be experiencing depression or anxiety and are advised to book an appointment with a primary care doctor. User experience testing found that the K10 was the most popular feature of the website.
3. The core (and the largest) component of Driving to Health is the self-help component which is called Healthy Activities. This component includes 31 individual activities designed to provide drivers with skills and techniques to reduce symptoms of stress, depression, and anxiety and to improve overall well-being. Activities include cognitive behavioral strategies, mindfulness and relaxation exercises, and physical health activities (see examples in Figure 2). These activities were selected by our team of experts on the basis of current mental health and occupational health literature [34-36]. Our previous research indicated that drivers use their mobile phone during the frequent breaks in their workday, but that they are unlikely to use computers at home. This finding informed our decision to include activities that drivers can do during the breaks in their working day and to not include longer, homework style activities.
4. The Find Help Now component provides phone numbers for crisis services, telephone counseling, and a link to a government website that can be used to find a doctor in the desired area.

### Aim

The aim of this study was to determine the feasibility and acceptability of *Driving to Health* and to assess its potential efficacy to increase mental health awareness, self-help behaviors, help-seeking intentions, and reduce psychological symptoms among taxi drivers. The results of this study will inform decisions around whether to proceed directly to a larger randomized controlled trial testing whether the intervention is effective or whether to modify the intervention and/or study design before proceeding to trial.

## Methods

### Design

This study was designed as a single-arm trial. Participants completed a suite of paper-based questionnaires at baseline, after which they were sent a link to the *Driving to Health* trial website. Participants were instructed to use *Driving to Health* as much or as little as they liked, in any way they liked, for 4 weeks. Because we wanted to replicate as much as possible how apps are used in real life, participants were not required to login upon each visit to the site. This meant that participants in the study could potentially share the link with others. After 4 weeks, participants completed a second suite of paper-based questionnaires. This study was approved by the Human Research Ethics Committee at the University of Melbourne (ID: 1749740).

### Participants

Taxi drivers working in metropolitan Melbourne, Australia, were recruited through advertisements at the Melbourne Airport Taxi Holding Yard and through the Facebook page of the Victorian Taxi Association. Drivers were eligible to participate in the study if they were aged between 21 and 70 years, had worked at least 4 driving shifts in the past month, and had an internet-enabled smartphone. Eligible drivers who expressed interest in participating were mailed out a study pack containing a plain language statement, consent form, baseline survey, and prepaid return envelope. Participants who returned both baseline and follow-up surveys were sent an Aus $150 gift card in recognition of their involvement in the study.

### Sample Size

There are no formal sample size criteria for feasibility studies as they are not designed to test for significant differences [37]. We aimed to recruit between 35 and 40 participants to this study, which would allow us to perform before and after testing (using 2-tailed paired *t* tests) to estimate trends in the data.

### Measures

Feasibility outcomes included recruitment, retention, and website use. Acceptability was determined by participants’ willingness to recommend *Driving to Health* to others, their anticipated use over the next 12 months, and their overall rating of the website. Potential efficacy was measured by self-reported mental health awareness, self-help behaviors and help-seeking intentions, and symptoms of stress, anxiety, and depression over time.

### Feasibility of Driving to Health

#### Recruitment and Retention

To determine the potential for *Driving to Health* to reach its target population, information on age, gender, education, relationship status, country of birth, year of settlement in Australia (if applicable), and main language spoken at home was obtained through the baseline questionnaire. Participants were also asked how many years they had worked as a taxi driver, the number of hours worked each week, and whether they worked night shift.

#### Use of the Website

Use of the website was examined using in-site analytic data. Google Analytics was used to measure the number of unique users, the duration of visits, and the number of pages viewed per session.

### Acceptability

Acceptability of *Driving to Health* was measured at a 4-week follow-up using 3 items from Section E of the Mobile App Rating Scale—User Version (uMARS) [38]. Each question was answered using a 5-point rating scale. Participants were asked (1) if they would recommend the website to people who might benefit from it (rated from 1 [*I would not recommend this website to anyone*] to 5 [*I would recommend this website to everyone*]); (2) how often they think they would use the website in the next 12 months (rated from 1 [*1-2 times*] to 5 [*more than 50 times*]); and (3) their overall rating of the website (rated from 1 [*one of the worst apps I have ever used*] to 5 [*one of the best apps I have ever used*]).

### Potential Efficacy

#### Perceived Impact of Driving to Health

Perceived impact of the website on awareness, attitudes, and behaviors was measured at the 4-week follow-up by 6 questions adapted from Section F of the uMARS [38]. Participants were asked if their use of *Driving to Health* had (1) increased their awareness of the importance of looking after their mental health; (2) increased their knowledge of mental health; (3) changed their attitudes to improving their mental health; (4) increased their intentions to look after their mental health; (5) encouraged them to seek help for their mental health; and (6) increased their behaviors for looking after their mental health. Each question was rated on a 5-point scale from 1 (*strongly agree*) to 5 (*strongly disagree*).

#### Help-Seeking Intentions

Intentions to seek help for mental health problems was measured using the General Help-Seeking Questionnaire (GHSQ) [39]. The GHSQ includes 3 categories of help: formal sources, informal sources, and self-help [39,40]. Formal sources of help include mental health professional (eg, psychologist, psychiatrist, social worker, and counselor), doctor/general practitioner (GP), religious leader (eg, Imam, Priest, Minister, and Rabbi), and phone helpline (eg, Lifeline). Informal sources of help include intimate partner (eg, wife/husband and girlfriend/boyfriend), friend, parent, family member/relative, and neighbor. Self-help options include internet support group and self-help group. Ratings for each item are made on a 7-point scale ranging from *extremely unlikely* (1) to *extremely likely* (7). Item scores within each of the 3 categories of help were used to calculate subscale scores. Studies on young people show that the GHSQ has reasonable reliability and validity [39].

#### Psychological Symptoms

Psychological distress was measured at baseline and at the 4-week follow-up using the short form of the Depression, Anxiety, and Stress Scale (DASS-21) [41]. The DASS-21 comprises 3 self-report subscales designed to measure symptoms of depression, anxiety, and tension/stress in the last week. Each of the 3 subscales includes 7 items and provides a valid measure of the dimensions of depression, anxiety, and stress [42]. Respondents use a 4-point scale to indicate how much each item applied to them with a range from 0 (*did not apply to me at all*) to 3 (*applied to me very much, or most of the time*). Scores for each item are summed to provide a subscale score. The DASS-21 has adequate construct validity and satisfactory to good reliability [42]. Ronk et al [43] reported that the minimal movement in scores required to indicate a clinically significant change (ie, a reliable and meaningful change) is 3.86 on the depression scale, 3.85 on the anxiety scale, and 4.90 on the stress scale.

### Data Analysis

Analysis was performed using STATA version 13.1 [44]. Complete case analysis was conducted on participants who returned both baseline and follow-up surveys. Variables were described using counts, frequencies, means, standard deviations, and 95% confidence intervals. Mean changes in scores on the GHSQ and the DASS-21 at baseline and 1 month were estimated using paired *t* tests. We used an alpha level of .05 for statistical tests.

## Results

### Recruitment and Sample Characteristics

**Figure 3:**
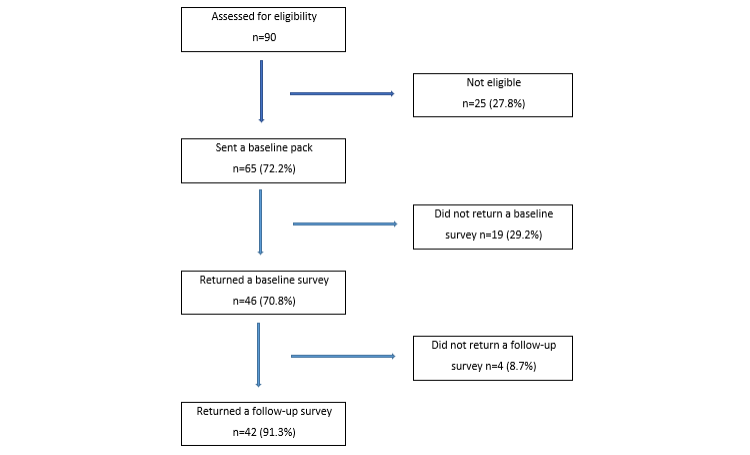
Flow of participants through the study.

A total of 46 drivers met the eligibility criteria, provided consent, and completed a baseline survey. Of those, 42 (91.3%) drivers returned completed follow-up surveys (Figure 3) with no demographic differences between those who did and did not. The average age of participants was 38.2 years (SD 9.8; range 22-57 years), and all participants were male (Table 1). Over a third (35.7%) of the sample had a university degree and 42.9% had a certificate qualification. Nearly all participants (40/42, 95%) were immigrants, with 51.3% (20/39) having settled permanently in Australia in the last 10 years. Participants were most likely to have been born in India (23/42, 54.8%), Pakistan (6/42, 14.3%), and the Horn of Africa (ie, Eritrea, Ethiopia, and Somalia) (6/42, 14.2%,). A total of 14 languages were spoken at home, including Punjabi (18/42, 42.9%), Urdu (5/42, 11.9%), English (5/42, 11.9%), Amharic (2/42, 4.8%), Somali (2/42, 4.8%), and Telugu (2/42, 4.8%).

**Table 1:** Sample characteristics.

Characteristics		Value, n (%)
Male (n=42)		42 (100)
**Age (n=40; years)**		
	20-29	9 (23)
	30-49	24 (60)
	50+	7 (18)
**Education (n=42)**		
	Before year 10	2 (5)
	Year 10 or equivalent	2 (5
	Year 12 or equivalent	5 (12)
	Certificate/diploma	18 (43)
	Bachelor’s degree	15 (36)
**Marital status (n=42)**		
	Never married	8 (19)
	Divorced	1 (2)
	Separated	2 (5)
	Married/de facto	31 (74)
**Living arrangements (n=40)**		
	Alone	2 (5)
	Spouse	29 (69)
	Children	22 (52)
	Parents	2 (5)
	Unrelated flatmate	8 (19)
	Other	3 (7)
**Country of birth (n=42)**		
	Australia	2 (5)
	Afghanistan	1 (2)
	Eritrea	2 (5)
	Ethiopia	2 (5)
	India	23 (55)
	Lebanon	2 (5)
	Pakistan	6 (14)
	Somalia	2 (5)
	Latvia	1 (2)
	Other	1 (2)
**Time in Australia (n=41; years)**		
	≤5	5 (13)
	6-10	15 (39)
	≥11	19 (49)

### Use

Analysis of Google Analytics for the study period showed that there were 89 unique users of *Driving to Health* (excluding members of the research team). Most users (85/89, 95.5%) accessed the website from Australia, with the remaining users accessing the website from Russia (2/89, 2.2%), India (1/89, 1.1%), and the United States (1/89, 1.1%). Overall, 91% of all visits were from a smartphone, and 9% of visits were from a desktop. Of the 85 Australia-based users, 55.3% (47/85) used the website once and 44.7% (38/85) were returning users. The average duration of a visit was 2 min and 31 seconds for single visit users and 4 min and 8 seconds for returning users. Single visit users and returning users both viewed an average of 4.94 pages per visit.

### Acceptability

A total of 24 % of participants (10/42) said that they would use the website more than 50 times in the next 12 months, and 40.5% (17/42) said that they would use it between 10 and 50 times (Table 2). Just over a quarter of the sample (11/42, 26.2%) said they would recommend *Driving to Health* to many people and 36% (15/42) said they would recommend it to everyone. More than two-thirds of participants (28/42, 67%) rated the website as above average.

**Table 2:** Acceptability of *Driving to Health*.

User rating of the app		Value, n (%)
**Would you recommend this app to people who might benefit from it? (n=42)**		
	I would not recommend this app to anyone.	4 (10)
	There are very few people I would recommend this app to	6 (14)
	There are a few people I would recommend this app to	6 (14)
	There are many people I would recommend this app to	11 (26)
	I would recommend this app to everyone	15 (36)
**How many times do you think you would use this app in the next 12 months? (n=42)**		
	None	2 (5)
	1-2 times	5 (12)
	3-10 times	8 (19)
	10-50 times	17 (41)
	More than 50 times	10 (24)
**What is your overall (star) rating of the app? (n=42)**		
	One of the worst apps I have used	2 (5)
	Below average	1 (2)
	Average	11 (26)
	Above average	21 (50)
	One of the best apps I have used	7 (17)

### Perceived Impact on Awareness, Behavior, and Intentions

Table 3 shows participants’ perceptions of the impact of *Driving to Health* on their mental health awareness, attitudes, and behaviors. Overall, 95% (40/42) of participants *agreed* or *strongly agreed* that using *Driving to Health* increased their awareness of looking after their mental health, and 85.7% (36/42) agreed or strongly agreed that it increased their knowledge of mental health (Table 3). More than three-quarters 33/42, 78.6%) of the sample agreed or strongly agreed that using the website changed their attitudes to improving their mental health and increased their mental health behaviors, while 70.7% (29/41) agreed or strongly agreed that it increased their intentions to look after their mental health. The perceived impact of *Driving to Health* was lowest for seeking help, where 63.4% (26/41) said that it encouraged them to seek help for their mental health problems, and 9.7% (4/41) disagreed or strongly disagreed with this statement.

**Table 3:** Perceived impact of *Driving to Health*.

Perceived impact		Value, n^a^ (%)
**Increased awareness of importance of looking after my mental health (n=42)**		
	Agree/strongly agree	40 (95)
	Neither agree nor disagree	1 (2)
	Disagree/strongly disagree	1 (2)
**Increased my knowledge of mental health (n=42)**		
	Agree/strongly agree	36 (86)
	Neither agree nor disagree	5 (12)
	Disagree/strongly disagree	1 (2)
**Changed my attitudes to improving mental health (n=42)**		
	Agree/strongly agree	33 (78)
	Neither agree nor disagree	7 (17)
	Disagree/strongly disagree	2 (5)
**Increased my intentions to look after my mental health (n=41)**		
	Agree/strongly agree	29 (71)
	Neither agree nor disagree	10 (24)
	Disagree/strongly disagree	2 (5)
**Encouraged me to seek help for my mental health (n=41)**		
	Agree/strongly agree	26 (63)
	Neither agree nor disagree	11 (27)
	Disagree/strongly disagree	4 (10)
**Increased my behaviors for looking after my mental health (n=42)**		
	Agree/strongly agree	32 (76)
	Neither agree nor disagree	8 (19)
	Disagree/strongly disagree	2 (5)

^a^Denominators vary because of missing data.

### Help-Seeking Intentions

At baseline, participants reported that they would be most likely to seek help from their partner, a GP, their parent, or a mental health professional (Table 4). They were least likely to identify neighbors and telephone helplines as sources of potential support. Only 2 participants (4.87%) said that it was likely that they would not seek help from anyone. No significant changes in help-seeking intentions were reported at follow-up.

### Psychological Symptoms

Scores increased on all 3 DASS-21 scales from baseline to follow-up, with paired *t* tests showing a statistically significant increase in anxiety symptoms (*P*=.02). However, as shown in Table 5, the magnitude of change did not meet the minimum movement required for reliable or meaningful change [43].

**Table 4:** General help-seeking questionnaire scores at baseline and follow-up.

Source of help	N^a^	Baseline, mean (SD)	Follow-up, mean (SD)	Difference	*P* value (*t* test; 2-sided)
Intimate partner	42	4.73 (2.23)	5.11 (1.99)	0.380	.33
Friend	41	3.97 (1.92)	4.36 (1.93)	0.390	.29
Parent	41	4.39 (2.32)	4.12 (2.29)	−0.268	.49
Relative	41	3.53 (2.19)	3.73 (1.91)	0.195	.58
Neighbor	42	2.00 (1.49)	2.30 (1.64)	0.309	.28
Mental health professional	42	4.28 (2.30)	4.19 (2.12)	−0.095	.81
Phone helpline	42	3.09 (2.09)	3.07 (2.06)	−0.023	.95
Doctor/general practitioner	42	4.71 (2.08)	4.88 (1.75)	0.166	.64
Religious leader	42	3.23 (2.10)	3.21 (2.05)	−0.023	.94
Internet support group	42	3.16 (2.11)	2.78 (1.85)	−0.308	.32
Self-help group	42	3.21 (1.98)	3.54 (1.91)	0.333	.36
Informal sources	39	18.53 (7.54)	20.17 (6.23)	1.64	.20
Formal sources	42	15.33 (7.16)	15.35 (6.17)	0.023	.98
Self-help	42	6.38 (3.66)	6.33 (3.06)	0.047	.94

^a^Denominators vary because of missing data.

**Table 5:** Depression, anxiety, and stress scale scores at baseline and follow-up.

Severity		n	Baseline			Follow-up			*t* test (*df*)	*P* value	Mean change	Minimal reliable change^a^
			%	Mean (SD)	95% CI	%	Mean (SD)	95% CI				
**Depression**		37	—^b^	6.15 (8.96)	3.17-9.15	—	7.08 (8.95)	4.09-10.06	1.22 (35)	.22	0.93	3.86
	Normal	28	75.7			67.6						
	Mild	5	13.5			13.5						
	Moderate	2	5.4			13.5						
	Severe	0	0			0						
	Extremely severe	2	5.4			5.4						
**Anxiety**		41	—	5.75 (6.08)	3.83-7.67	—	7.80 (8.19)	5.21-10.39	2.45 (39)	.02	2.05	3.85
	Normal	25	61.0			56.1						
	Mild	9	22.0			12.2						
	Moderate	2	4.9			9.8						
	Severe	3	7.3			12.2						
	Extremely severe	2	4.9			9.8						
**Stress**		34	—	6.88 (6.71)	4.53-9.22	—	8.58 (6.98)	6.14-11.02	1.79 (32)	.08	1.7	4.90
	Normal	34	81.0			69.1						
	Mild	2	4.8			11.9						
	Moderate	3	7.1			4.8						
	Severe	0	0			0						
	Extremely severe	3	7.1			14.3						

^a^Change in scores required to indicate a reliable and meaningful change, as defined by Ronk et al [43].

^b^Not applicable.

## Discussion

### Principal Findings

*Driving to Health* is the world’s first eHealth intervention designed for the taxi industry, which comprises a large population of mostly immigrant men. The high completion rate achieved in this study, combined with the diversity of participants recruited (participants born in 10 different countries speaking 14 different languages), provides strong support for the feasibility and acceptability of *Driving to Health* amongst its target population.

Almost two-thirds of participants strongly endorsed recommending the website to others. This finding was reflected by the fact that although only 46 drivers were sent the website link there were almost twice this number of unique users, suggesting that participants shared the link with others. Website analytics showed that just under half (45%) of all users were returning users, which is a retention rate similar to the average 1-month retention rate of 43% for mobile apps [45]. Because we did not require users to logon at each session, we were not able to track website use for individual study participants. Thus, average usage statistics may have been either diluted or exaggerated by the inclusion of users who were not part of the study. Self-reported intentions to use the website over the next 12 months showed a much higher level of engagement, with 41% of study participants saying they would use it between 10 and 50 times and 24% saying they would use it more than 50 times. Although this suggests that the average use statistics were diluted, rather than exaggerated, by the inclusion of nonstudy participants, it is also possible that the discrepancy between objective use and subject reports of intention to use the app is because of socially desirable responding. The average time spent using the website each session (4 min) was comparable with other eHealth interventions [46,47] and is roughly what we expected given the median duration of exercises included in the Healthy Activities section (5 min). This supports our decision to include short activities that can be completed in between jobs. It is worth noting, however, that the minimum *dose* of *Driving to Health* that could be considered effective is unclear. Although this study was not powered to investigate dose-response relationships, this is an avenue worthy of further exploration and may lead to recommendations for future users about how to get the most out of the site.

Results from the uMARS regarding the potential effectiveness of *Driving to Health* in increasing awareness, teaching self-help skills, and promoting help-seeking were very positive. Over 95% of participants agreed that using the site increased their awareness of mental health, and 86% agreed that it increased their knowledge of mental health. Over three-fourths agreed that *Driving to Health* improved their health behaviors, and 63% said it encouraged them to seek help for their mental health if they needed it. However, the GHSQ did not show an increase in intentions to seek help from either formal or informal sources.

Similarly, the self-reported acceptability and impact of the website were not reflected in self-reported psychological symptoms. In fact, there was a small increase in anxiety scores on the DASS-21 over the 4-week study period. We are unable to identify any obvious mechanism in the website that would contribute to this, and although we cannot verify it, it is possible that the slight increase in anxiety is attributable to some underreporting of symptoms at baseline and a subsequent correction in this at follow-up. Consistent with this explanation is the relatively low level of psychological symptoms at baseline combined with high rates of increased awareness and knowledge about mental health during the study period. Previous research has shown that increases in awareness and knowledge of mental health are associated with a reduction in stigmatizing attitudes and a subsequent increased willingness to disclose mental health symptoms [48].

### Strengths and Limitations

That over 90% of the participating drivers completed a follow-up survey is a substantial strength of this study. This suggests that taxi drivers are interested in health interventions designed specifically for them, that the study methods were acceptable to this culturally and linguistically diverse population, and that a mobile phone platform is feasible for such interventions. Another strength of the study was measuring website use via objective data obtained through Google Analytics rather than relying on self-reported use, which previous studies have done. A weakness in our methodology was not requiring participants to login to the website. Although this approach enabled participants to access the site easily and to share the link with others, which provides an indication of acceptability, it meant we could not limit our usage analysis to study participants alone or identify whether users were unique (eg, study participants may have accessed the site from more than one device). It also meant that we could not link website use with DASS-21 scores and explore the relationship between the frequency of use and changes in psychological symptoms. In addition, the relatively low rates of baseline psychological symptoms may have resulted in a floor effect, where the potential for improvement was minimal, and may suggest either that drivers with the poorest mental health did not take part or that participants underreported their symptoms at baseline. This issue requires further investigation as both possibilities here pose different challenges to a future rollout of the *Driving to Health* app. It should also be noted that all the study measures were in English, whereas 95% of participants were from a non-English speaking background. It is unknown what influence this may have had on the results. Furthermore, the expression and understanding of psychological distress may be culturally bound, and our measures may not accurately capture the experience of the different cultural groups that comprise our sample. The 4-week time frame may also have been too short to produce meaningful changes in psychological symptoms. Finally, because we did not include a validated mental health literacy scale in the study, we cannot confirm our suspicion that the observed increase in psychological symptoms was because of an increase in mental health literacy.

### Implications and Further Research

Hundreds of thousands of people work as taxi drivers across the world. They are a vulnerable workforce who have been neglected by occupational health interventions. This study shows that urban taxi drivers are receptive to mobile health interventions designed specifically for them and are willing to participate in related research. Overall, the results suggest that a randomized controlled trial aimed at investigating the effectiveness of *Driving to Health* is feasible. However, the findings also indicate that several modifications to the study design are needed before progressing to a trial. In particular, the trial should include a validated measure of mental health literacy, require participants to login to the site so that we can track the relationship between individual use and outcomes, and use a different measure of psychological distress. The trial should also encompass a longer period. If found to be effective, *Driving to Health* could be scaled to other professional drivers, including delivery drivers and truck drivers. It could also be expanded to include physical health conditions and be integrated with peer or other industry support services to enhance its impact on mental health.

### Conclusions

Taxi drivers’ reports of the impact of *Driving to Health* indicate that it is a promising intervention, especially in terms of increasing mental health awareness and knowledge, which are precursors to seeking help for mental health problems [49,50]. We anticipate that ongoing refinement of the intervention and research into its efficacy will contribute to improvements in mental health behaviors and outcomes for a vulnerable occupational group.

## Abbreviations

DASS-21: Depression, Anxiety, and Stress scale
eHealth: electronic health
GHSQ: General Help-Seeking Questionnaire
GP: general practitioner
K10: 10-item Kessler Psychological Distress Scale
uMARS: user version of the Mobile App Rating Scale

## References

[1] Davidson S, Wadley G, Reavley N, Gunn J, Fletcher S (2018). Psychological distress and unmet mental health needs among urban taxi drivers: a cross-sectional survey. Aust N Z J Psychiatry.

[2] Dosen I (2016). Parliament of Victoria.

[3] Gany FM, Gill PP, Ahmed A, Acharya S, Leng J (2013). 'Every disease…man can get can start in this cab': focus groups to identify south Asian taxi drivers' knowledge, attitudes and beliefs about cardiovascular disease and its risks. J Immigr Minor Health.

[4] Mayhew C (2000). Australian Institute of Criminology.

[5] Burgel BJ, Gillen M, White MC (2012). Health and safety strategies of urban taxi drivers. J Urban Health.

[6] (2007). Taxi-Library.

[7] Stewart N, Ferré-Sadurní L The New York Times.

[8] Gany F, Gill P, Baser R, Leng J (2014). Supporting South Asian Taxi Drivers to Exercise through Pedometers (SSTEP) to decrease cardiovascular disease risk. J Urban Health.

[9] KPMG (2011). Australian Taxi Industry Association: Demographic analysis of the Australian taxi industry.

[10] (2004). Schaller Consulting.

[11] Xu L (2012). Government of Canada.

[12] Sarría-Santamera A, Hijas-Gómez AI, Carmona R, Gimeno-Feliú LA (2016). A systematic review of the use of health services by immigrants and native populations. Public Health Rev.

[13] (2012). Beyond Blue.

[14] Kokanovic R, Dowrick C, Butler E, Herrman H, Gunn J (2008). Lay accounts of depression amongst Anglo-Australian residents and East African refugees. Soc Sci Med.

[15] Reavley NJ, Jorm AF (2011). Stigmatizing attitudes towards people with mental disorders: findings from an Australian National Survey of Mental Health Literacy and Stigma. Aust N Z J Psychiatry.

[16] Office of the Surgeon General (US), Center for Mental Health Services (US), National Institute of Mental Health (US) (2001). Mental Health: Culture, Race, and Ethnicity—A Supplement to Mental Health: A Report of the Surgeon General.

[17] Davidson S, Wadley G, Reavley N, Gunn J, Russon P (2016). The driving for change project. Identifying the mental health needs of Melbourne Taxi Drivers: Draft report.

[18] Firth J, Torous J, Nicholas J, Carney R, Pratap A, Rosenbaum S, Sarris J (2017). The efficacy of smartphone-based mental health interventions for depressive symptoms: a meta-analysis of randomized controlled trials. World Psychiatry.

[19] Firth J, Torous J, Nicholas J, Carney R, Rosenbaum S, Sarris J (2017). Can smartphone mental health interventions reduce symptoms of anxiety? A meta-analysis of randomized controlled trials. J Affect Disord.

[20] Donker T, Petrie K, Proudfoot J, Clarke J, Birch M, Christensen H (2013). Smartphones for smarter delivery of mental health programs: a systematic review. J Med Internet Res.

[21] Mohr DC, Burns MN, Schueller SM, Clarke G, Klinkman M (2013). Behavioral intervention technologies: evidence review and recommendations for future research in mental health. Gen Hosp Psychiatry.

[22] Payton FC, Yarger LK, Pinter AT (2018). Text mining mental health reports for issues impacting today's college students: qualitative study. JMIR Ment Health.

[23] Miner A, Kuhn E, Hoffman JE, Owen JE, Ruzek JI, Taylor CB (2016). Feasibility, acceptability, and potential efficacy of the PTSD Coach app: a pilot randomized controlled trial with community trauma survivors. Psychol Trauma.

[24] Han M, Lee E (2018). Effectiveness of mobile health application use to improve health behavior changes: a systematic review of randomized controlled trials. Healthc Inform Res.

[25] Taylor-Rodgers E, Batterham PJ (2014). Evaluation of an online psychoeducation intervention to promote mental health help seeking attitudes and intentions among young adults: randomised controlled trial. J Affect Disord.

[26] Musiat P, Tarrier N (2014). Collateral outcomes in e-mental health: a systematic review of the evidence for added benefits of computerized cognitive behavior therapy interventions for mental health. Psychol Med.

[27] van Gemert-Pijnen JE, Nijland N, van Limburg M, Ossebaard HC, Kelders SM, Eysenbach G, Seydel ER (2011). A holistic framework to improve the uptake and impact of eHealth technologies. J Med Internet Res.

[28] Payton FC (2015). Cultures of participation-for students, by students. Info Sys J.

[29] Flesch R (1948). A new readability yardstick. J Appl Psychol.

[30] Kincaid JP, Fishburne RP, Rogers RL, Chissom BS ucf stars - University of Central Florida.

[31] Kessler R, Andrews G, Colpe L, Hiripi E, Mroczek D, Normand S, Walters E, Zaslavsky A (2002). Short screening scales to monitor population prevalences and trends in non-specific psychological distress. Psychol Med.

[32] (2005). Harvard Medical School.

[33] (2016). The Primary Mental Health Care Minimum Data Set.

[34] Joyce S, Shand F, Tighe J, Laurent SJ, Bryant RA, Harvey SB (2018). Road to resilience: a systematic review and meta-analysis of resilience training programmes and interventions. BMJ Open.

[35] Bourne EJ (2015). The Anxiety and Phobia Workbook. Sixth Edition.

[36] Healthier Workplace WA.

[37] Eldridge SM, Lancaster GA, Campbell MJ, Thabane L, Hopewell S, Coleman CL, Bond CM (2016). Defining feasibility and pilot studies in preparation for randomised controlled trials: development of a conceptual framework. PLoS One.

[38] Stoyanov SR, Hides L, Kavanagh DJ, Wilson H (2016). Development and validation of the user version of the Mobile Application Rating Scale (uMARS). JMIR Mhealth Uhealth.

[39] Wilson C, Deane F, Ciarrochi J, Rickwood D (2005). Measuring help-seeking intentions: properties of the General Help-Seeking Questionnaire. Can J Couns.

[40] Rickwood D, Thomas K, Bradford S (2012). Sax Institute.

[41] Lovibond SH, Lovibond PF (1995). Trove - National Library of Australia.

[42] Henry JD, Crawford JR (2005). The short-form version of the Depression Anxiety Stress Scales (DASS-21): construct validity and normative data in a large non-clinical sample. Br J Clin Psychol.

[43] Ronk FR, Korman JR, Hooke GR, Page AC (2013). Assessing clinical significance of treatment outcomes using the DASS-21. Psychol Assess.

[44] Stata: Software for Statistics and Data Science.

[45] Armour B (2018). Clearbridge Mobile.

[46] Baskerville NB, Struik LL, Dash D (2018). Crush the crave: development and formative evaluation of a smartphone app for smoking cessation. JMIR Mhealth Uhealth.

[47] Song MJ, Ward J, Choi F, Nikoo M, Frank A, Shams F, Tabi K, Vigo D, Krausz M (2018). A process evaluation of a web-based mental health portal (WalkAlong) using google analytics. JMIR Ment Health.

[48] Reavley NJ, Jorm AF (2014). Willingness to disclose a mental disorder and knowledge of disorders in others: changes in Australia over 16 years. Aust N Z J Psychiatry.

[49] Rüsch N, Evans-Lacko SE, Henderson C, Flach C, Thornicroft G (2011). Knowledge and attitudes as predictors of intentions to seek help for and disclose a mental illness. Psychiatr Serv.

[50] Bonabi H, Müller M, Ajdacic-Gross V, Eisele J, Rodgers S, Seifritz E, Rössler W, Rüsch N (2016). Mental health literacy, attitudes to help seeking, and perceived need as predictors of mental health service use: a longitudinal study. J Nerv Ment Dis.

